# Architecture Design of a Convolutional Neural Network Accelerator for Heterogeneous Computing Based on a Fused Systolic Array

**DOI:** 10.3390/s26020628

**Published:** 2026-01-16

**Authors:** Yang Zong, Zhenhao Ma, Jian Ren, Yu Cao, Meng Li, Bin Liu

**Affiliations:** School of Information Science and Engineering, Shenyang University of Technology, Shenyang 110870, China; zongyang06@126.com (Y.Z.); 18018383183@163.com (Z.M.); renj@sut.edu.cn (J.R.); cy18341622925@126.com (Y.C.); 13998284051@163.com (B.L.)

**Keywords:** convolutional neural network, hardware accelerator, systolic array, heterogeneous computing, operator fusion

## Abstract

Convolutional Neural Networks (CNNs) generally suffer from excessive computational overhead, high resource consumption, and complex network structures, which severely restrict the deployment on microprocessor chips. Existing related accelerators only have an energy efficiency ratio of 2.32–6.5925 GOPs/W, making it difficult to meet the low-power requirements of embedded application scenarios. To address these issues, this paper proposes a low-power and high-energy-efficiency CNN accelerator architecture based on a central processing unit (CPU) and an Application-Specific Integrated Circuit (ASIC) heterogeneous computing architecture, adopting an operator-fused systolic array algorithm with the YOLOv5n target detection network as the application benchmark. It integrates a 2D systolic array with Conv-BN fusion technology to achieve deep operator fusion of convolution, batch normalization and activation functions; optimizes the RISC-V core to reduce resource usage; and adopts a locking mechanism and a prefetching strategy for the asynchronous platform to ensure operational stability. Experiments on the Nexys Video development board show that the architecture achieves 20.6 GFLOPs of computational performance, 1.96 W of power consumption, and 10.46 GOPs/W of energy efficiency ratio, which is 58–350% higher than existing mainstream accelerators, thus demonstrating excellent potential for embedded deployment.

## 1. Introduction

Object Detection (OD) is a prominent research direction in computer vision and pattern recognition, aiming to accurately identify and localize multiple objects belonging to specific categories within images [1,2]. With the advancement of deep learning, the accuracy and efficiency of object detection have been significantly enhanced. Over the past decade, CNNs have found widespread application in visual tasks owing to their powerful feature extraction capabilities and efficient computational performance [3,4], thus becoming one of the mainstream methods in the field of OD. Several representative algorithms have emerged, such as Faster R-CNN [5], YOLO [6], ResNetX [7], and MobileNets [8]. Among these, the YOLO-series algorithms have achieved remarkable results across various OD tasks due to their superior performance [9,10,11].

As the complexity and performance of object detection algorithms continue to improve and model sizes gradually increase, this poses challenges for the implementation of neural network chips [12]. For instance, the YOLOv5 model series, characterized by its compact architecture and ease of deployment, has a parameter size of 46.5 MB for YOLOv5l; the later released YOLOv5x, by contrast, increases to 86.7 MB of parameters and a computational load of 205.7 GFLOPs [13]. The multi-layer feature fusion architecture and Spatial Pyramid Pooling-Fast (SPPF) operation in YOLOv5 involve extensive matrix multiplication and data interaction, which demand substantial computational units and memory resources for chip implementation to ensure operational efficiency [14]. Simultaneously, the massive model parameters result in significant time and hardware resource consumption during data movement and scheduling. These challenges restrict the application of neural networks to high-power, high-performance devices such as desktop computers or industrial control systems, thereby limiting the technological advancement of AI in edge computing and low-power scenarios [15,16].

To address the above circumstances, this paper focuses on resolving the deployment challenges of convolutional neural networks in resource-constrained scenarios. Accordingly, a heterogeneous computing convolutional neural network accelerator architecture based on the fused systolic array algorithm is designed, where the YOLOv5n network is used as the application benchmark. The main research work and contributions are as follows: (1) Operator fusion is achieved by integrating the Batch Normalization (BN) layer, Convolution (CONV) layer, and activation function. A high-performance convolutional accelerator algorithm based on the fused-operator systolic array is proposed. An attention mechanism is incorporated to mitigate the reduction in recognition accuracy induced by operator fusion, and modeling, simulations, and verification are performed in MATLAB (R2022a). (2) A low-power CPU based on the RISC-V instruction set is designed using an ICB bus. This CPU supports integration with convolutional accelerator units, pooling accelerator units, and cache modules, enabling the construction of a high-energy-efficiency CPU + ASIC heterogeneous computing platform. (3) The accelerator architecture is tested on the Digilent Nexys Video board platform (Digilent, Inc., Pullman, DC, USA) equipped with a Xilinx XCA7A35T Field Programmable Gate Array (FPGA). Test results using the BIT-Vehicle vehicle detection dataset demonstrate that operator fusion achieves significant computational speedup with minimal accuracy loss. The heterogeneous computing accelerator based on the fused systolic array achieves a throughput of 20.6 GOPS at a power consumption of only 1.96 W. Its energy efficiency significantly outperforms that of CPU and GPU deployments for convolutional neural networks, while its computational speed surpasses that of comparable accelerators validated on FPGAs.

The organization of this paper is as follows: Section 1 provides a review of the development of CNNs and the research content of this paper. Section 2 elaborates on the reasons for network selection, introduces the corresponding model, and identifies the problems to be solved. Section 3 presents a detailed description of the principles and schemes for accelerator design. Section 4 demonstrates the verification schemes and results of the accelerator. Finally, Section 5 offers a summary and discussion of the full paper.

## 2. Related Work

### 2.1. Object Detection Network

You Only Look Once (YOLO) is a widely adopted object detection algorithm characterized by high accuracy and low latency, enabling real-time object detection [17,18]. Since its debut in 2015, it has undergone multiple iterations and optimizations, with successive versions continuously improved. Its detection performance and efficiency have been significantly enhanced, providing valuable references for research and engineering applications in related fields [19,20].

At the hardware implementation level, the mainstream network models adopted include YOLOv3–Tiny [21], YOLOv4–Tiny [22], YOLOv5n [23], and YOLOv8n [24]. Their network parameters, model sizes, and release times are shown in Table 1. In comparison, YOLOv5n has the following significant advantages: First, it has a small number of model parameters and adopts an extremely lightweight design [25], which can effectively reduce the occupation of hardware computing resources and storage resources, allowing it to adapt to the resource-constrained deployment environment in integrated circuit systems. Second, the model architecture is simple, allowing it to achieve excellent real-time inference performance and a fast detection speed [26], making it suitable for integrated circuit-related application scenarios with strict requirements on real-time performance. Based on the above advantages, this study finally selects the YOLOv5n network as the core detection model to carry out relevant research work.

### 2.2. YOLOv5n Algorithm Model and Attention Module

**Figure 1:**
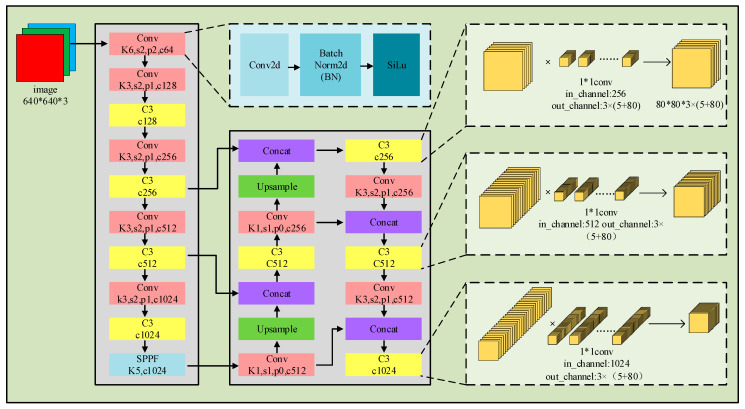
Operational diagram of YOLOv5n. The color-coded blocks represent different functional modules: pink for the standard Convolutional module (including Conv2d, Batch Normalization, and SiLu activation); yellow for the C3 feature extraction module; purple for the Concatenation operation (feature fusion); green for the Upsampling layer; and light blue for the SPPF module.

The main architecture of the YOLOv5n network is illustrated in Figure 1 and is derived from the original work of the authors. It consists of fundamental operations including CONV, BN, the Sigmoid Linear Unit (SILU), Max Pooling (MaxPool), and feature concatenation (CONCAT) [27]; its input is a 640 × 640 3-channel RGB image, and the backbone implements feature extraction using Conv layers annotated as “Conv Kx,sx,px,cx”, where K, s, p, and c denote the kernel size, stride, padding and output channel number, respectively, paired with C3 modules annotated as “C3 cx”, with cx representing the output channel number, while multi-scale feature aggregation is achieved via the “SPPF K5,c1024” module. In the neck, features from different backbone levels are fused through Upsample and Concat operations, with the fused features processed by reused Conv layers and C3 modules; the detection head employs 1 × 1 convolution layers annotated as “1*1conv” to adjust channel numbers, outputting channels as “3 × (5 + 80)”, where 3 stands for prediction boxes per grid, 5 corresponds to box coordinates and confidence, and 80 refers to object classes in the COCO dataset, to generate multi-scale detection feature maps such as 80 × 80. Additionally, general modules including Conv2d, Batch Norm2d, and SiLu, which correspond to convolution operations, batch normalization, and activation functions, respectively, are integrated to support the network’s feature learning and detection tasks.

**Figure 2:**
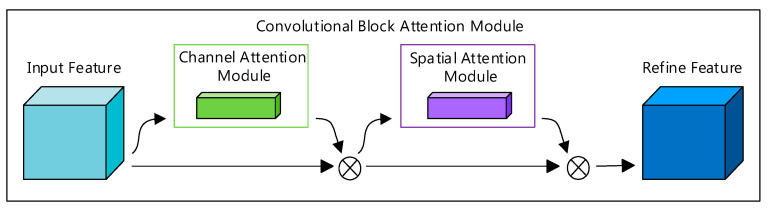
Convolutional Block Attention Module. The diagram illustrates the sequential application of attention modules. Light blue represents the intermediate input feature map; green denotes the Channel Attention Module (CAM) which focuses on ‘what’ is meaningful; purple denotes the Spatial Attention Module (SAM) which focuses on ‘where’ is informative; and dark blue represents the final refined feature map.

The Convolutional Block Attention Module (CBAM) aims to overcome the limitations of traditional convolutional neural networks in feature extraction. Through comparative analyses, researchers have demonstrated significant performance improvements by integrating CBAMs into classic architectures such as ResNet and MobileNet [28]. The CBAMs comprises two submodules: the Channel Attention Module (CAM) and the Spatial Attention Module (SAM) [29]. The Channel Attention Module enhances feature map representation by learning the importance of each channel, while the Spatial Attention Module strengthens spatial feature representation by learning the importance of each spatial location. The integration of CBAMs improves model performance by enabling the network to focus more on target object recognition, thereby enhancing model interpretability. CBAMs can be incorporated into any convolutional layer to boost feature extraction capabilities, ultimately improving the model’s recognition accuracy [30]. The CBAM, which is from the original work of the authors, is illustrated in Figure 2.

### 2.3. Analysis of Existing Problems

The YOLOv5n model itself exhibits high computational and control complexity, requiring a large number of arithmetic and control operations to complete a single inference task. In the context of accelerator design, such repetitive control operations are typically implemented using state machine groups or similar control mechanisms; however, an overly hierarchical structure can compromise the performance and reliability of the overall design [31]. Meanwhile, BN and SILU operations involve a large number of nonlinear computational operations, which are difficult to implement directly on hardware accelerators [32]. Moreover, the adoption of hardware-friendly algorithms for these operations tends to result in losses of model accuracy. In addition, convolutional operations impose a heavy computational burden on a single inference task, while feature fusion operations within the network lead to temporal discontinuities in the output data of intermediate layers [33]. These issues collectively further exacerbate the challenges in accelerator design.

To address the aforementioned problems, current hardware accelerators targeting the YOLOv5 network mainly adopt two technical approaches: model optimization and accelerator architecture improvement [34,35]. Reference [36] enhances accelerator performance by optimizing model structures and deployment quantization strategies. Specific measures include replacing standard convolutions with depthwise separable convolutions to achieve lightweighting of the YOLOv5s model and substituting the original backbone network with a MobileNet architecture to further reduce computational complexity and the parameter count. Reference [37] proposes a heterogeneous computing architecture, where software design is employed to handle complex and resource-intensive scheduling modules, while the convolutional acceleration unit is fully controlled by the CPU. By leveraging the superior scalability of the RISC-V instruction set, this architecture constructs a set of acceleration library functions and connects the accelerator to the CPU as a coprocessor via the EAI interface. The advantages of this scheme lie in transforming the accelerator design into a coprocessor form, which features simplified programming, high code density, and significant resource savings. Meanwhile, on-chip cache units can reduce data redundancy, improve data transmission efficiency, and thereby enhance the computational speed of the accelerator at the hardware level.

Nevertheless, none of the aforementioned accelerator design schemes have fully exploited the potential for performance acceleration. For instance, the model lightweighting strategy in Reference [36] comes at the cost of sacrificing recognition accuracy and fails to fully utilize the technical advantages of ASICs in large-scale matrix operations. Reference [37] transforms the hardware-driven design paradigm into a software-assisted one; although it improves data utilization through a caching mechanism, its accelerator architecture still suffers from resource wastage issues.

## 3. Accelerator Architecture Design

### 3.1. Convolution Layer and Batch Normalization Layer Fusion

**Figure 3:**
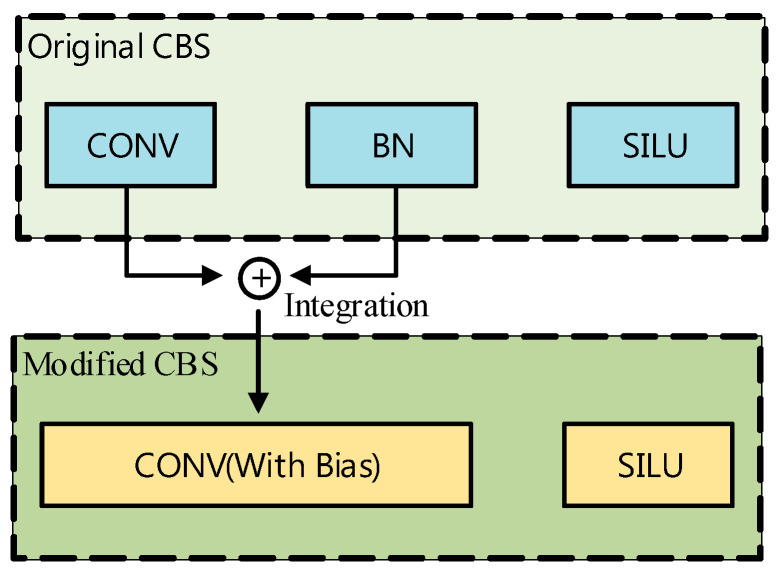
Schematic diagram of CONV-layer and BN-layer integration. The light blue blocks denote the individual components (CONV, BN, and SILU) in the original CBS module during the training phase. The yellow blocks represent the fused components in the modified CBS module, where BN parameters are mathematically integrated into the convolution layer (CONV with Bias) to enhance inference efficiency.

When calculating the number of parameters in the YOLOv5n model, the CONV, BN, and SILU layers must be taken into account, among which CONV and BN exhibit strong interdependencies. Therefore, computational optimization should first integrate the CONV and BN layers to reduce the computational load, thereby lowering the consumption of hardware resources. This optimization strategy is illustrated in Figure 3, which is derived from the original work of the authors.

Assuming that in a single convolution operation, the input convolution kernel parameter is denoted as *ω* and the input feature map parameter is denoted as *x*, the convolution output *y_conv_* can be calculated according to Equation (1).

(1) $$y_{conv}=\omega \cdot x+b$$

We then substitute the one-pass calculation formula of the aforementioned convolutional layer into the calculation formula of the BN layer [38], and the resulting expression is shown in Equation (2).

(2) $$\begin{array}{ll} BN_{\gamma ,\beta }\left(x\right) & =\gamma \frac{\omega \cdot x+b-\mu_{B}}{\sqrt{\sigma_{B}^{2}+\epsilon }}+\beta \\ & =\frac{\gamma \cdot \omega }{\sqrt{\sigma_{B}^{2}+\epsilon }}\cdot x+\frac{\gamma }{\sqrt{\sigma_{B}^{2}+\epsilon }}\cdot \left(b-\mu_{B}\right)+\beta \end{array}$$

Equation (3) describes the normalization and scaling process of network weights, while Equation (4) defines the normalization and shifting process of bias terms [39]. Substituting both equations into Equation (2) yields Equation (5).

(3) $$\hat{\omega }=\frac{\gamma \cdot \omega }{\sqrt{\sigma_{B}^{2}+\epsilon }}$$

(4) $$\hat{b}=\frac{\gamma }{\sqrt{\sigma_{B}^{2}+\epsilon }}\cdot \left(b-\mu_{B}\right)+\beta$$

(5) $$BN_{\gamma ,\beta }\left(x\right)=\hat{\omega }\cdot x+\hat{b}$$

As shown in Equation (5), the computations of the CONV and BN layers can be fused into a single CONV-like operation, which is termed the CBN operator. The computational complexity of this operator is equivalent to that of a convolutional operation with a bias term, and this fusion reduces the model’s computational load to approximately 60% of that of the original model.

### 3.2. Fused Systolic Array Algorithm

The convolution formula is presented below [39]. Specifically, the convolution kernel traverses the input feature map in a row-wise order with a stride of 1 pixel. During the traversal, it performs multiply–accumulate (MAC) operations on the covered local data. For the structural matching of convolution operations, the depth of each convolution kernel is consistent with that of the input feature map layer, while the number of convolution kernels in each layer is equal to the depth of the output feature map layer.

(6) $$Q_{x,y,n}=\sum_{d}\sum_{j}\sum_{i}\left(C_{i,j,d,n}\times P_{x+i,y+j,d}\right)+B_{n}$$

Here, *Q*_*x*,*y*,*n*_ denotes a pixel in the output feature map layer, *C*_*i*,*j*,*d*,*n*_ represents the parameters of the convolution kernel, and *P*_*x*+*i*,*y*+*j*,*d*_ corresponds to the pixel coordinates in the input feature map layer. To clarify the definition of each dimension parameter, *x* and *y* are the horizontal and vertical coordinates of pixels in the feature map layer, respectively; *n* denotes the depth coordinate of the output feature map layer; *d* represents the depth coordinate of the input feature map layer; and *i* and *j* are the horizontal and vertical coordinates of the convolution kernel parameters, respectively. From the parameter correlation and computation process described above, it can be observed that convolution operations inherently exhibit parallel computing characteristics.

The systolic array enhances the parallelism of convolution operations by constructing a rectangular processing array. It consists of 224 processing elements (PEs) arranged in a 16 × 16 configuration, with serial interconnections between the elements. Each processing element comprises a multiplier, an adder, and three sets of registers—where the registers are used to store pass-through parameters and intermediate computation results. Specifically, each column of the array takes in data from a convolution kernel, while each row inputs the corresponding input feature map data for that kernel. The computation proceeds in the downward and rightward directions, and the output of each processing element in the array corresponds to a single pixel in the output feature map layer. Taking a 3 × 3 systolic array as an example, Equations (7) and (8) illustrate the computational mechanism of the array and the operating principle of a single processing element, respectively, with both formulations based on the conventional matrix multiplication operation.

(7) $$\left[\begin{array}{ccc} PE_{00} & PE_{01} & PE_{02}\\ PE_{10} & PE_{11} & PE_{12}\\ PE_{20} & PE_{21} & PE_{22} \end{array}\right]=\left[\begin{array}{ccc} P_{00} & P_{01} & P_{02}\\ P_{10} & P_{11} & P_{12}\\ P_{20} & P_{21} & P_{22} \end{array}\right]*\left[\begin{array}{ccc} C_{00} & C_{01} & C_{02}\\ C_{10} & C_{11} & C_{12}\\ C_{20} & C_{21} & C_{22} \end{array}\right]$$

(8) $$PE_{00}=P_{00}\times C_{00}+P_{01}\times C_{10}+P_{02}\times C_{20}$$

In this context, PE denotes the output of a processing element within the systolic array, *P* represents the horizontally input feature map pixels, and *C* signifies the vertically input convolution kernel parameters. The final output of each systolic array corresponds to the result of a complete convolution operation, and the consistent accumulation operations within the array facilitate streamlined processing of the convolution task.

**Figure 4:**
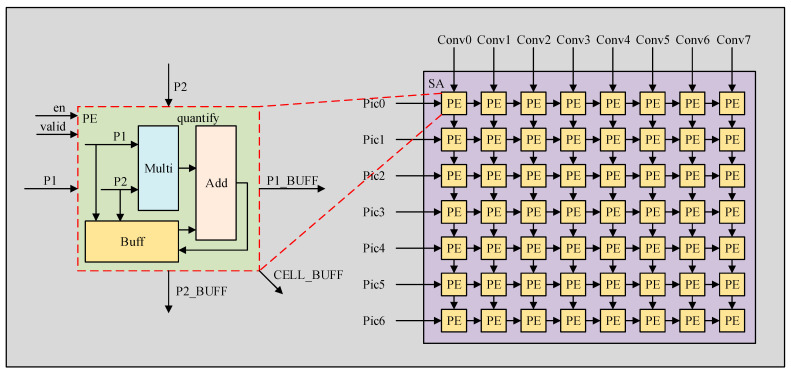
Fused systolic array architecture. The green dashed box illustrates the micro-architecture of a PE, featuring a Multiplier (blue), a local Buffer (orange), and an Adder (pink) for quantification. The purple region denotes the overall SA layout, where yellow blocks represent the grid-mapped PE nodes interconnected for data flow (Pic and Conv).

By incorporating the BIAS parameter into Equation (8), we obtain Equation (9). Considering the flushing characteristics of the systolic array, an additional set of adders (denoted as *B*) is added to the right and bottom sides of the array. After completing a convolution calculation and clearing the intermediate data, the results of the CBN operator can be continuously output at the adders (*B*). This design ultimately defines the integrated systolic array structure, which is illustrated in Figure 4.

(9) $$CBN_{00}=P_{00}\times C_{00}+P_{01}\times C_{10}+P_{02}\times C_{20}+BIAS_{0}$$

Building upon the aforementioned foundation, we explored the secondary operator fusion technique by combining the CBN operator with the SILU function to derive a linear iterative scheme. This approach achieves compression for approximately 80% of the computational operations during network inference, along with minimal additional hardware resource consumption.

### 3.3. Pooled Accelerator Design

Pooling operation replaces the value of a point in a two-dimensional image layer with the maximum value within a matrix centered on that point. Taking (*m*, *n*) as the vertex and *k* as the radius, a *k* × *k*-sized matrix, *A*, is constructed. Matrix *A* contains *k* × *k* elements in total; let the value of the top-left element be *x_i_*, and let the maximum value in the matrix be *x_max_*. The result of the pooling calculation is given by Equation (10).

(10) $$x_{i}=x_{\max}$$

As shown in Equation (10), the pooling operation requires accessing *k* × *k* elements, which imposes substantial memory access pressure on the system. To alleviate such pressure, a data-prefetching strategy is adopted. Meanwhile, data is transmitted through a cascaded pipeline with progressive discarding of lower-order bits, thereby ensuring that no data blocking occurs in the acceleration unit. Each pooling segment is defined as shown in Equation (11), where *n* denotes the pooling kernel index, *d* represents the kernel size, and *Z* denotes the image width.

(11) $$\left[n,n+d\right]\;(n\in N,n\in [0,Z])$$

**Figure 5:**
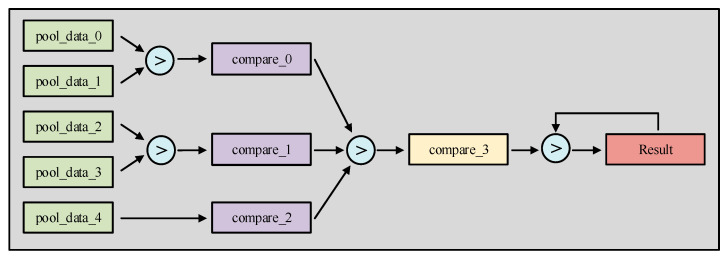
Flow-through batch acceleration unit. Different colors represent the functional stages of the pooling data pipeline: green blocks for input pooling data, purple and yellow blocks for intermediate comparison results at different stages, and the red block for the final output result. The light blue circles indicate the comparison logic units.

Next, the elements within each pooling segment are compared pairwise. Combined with the corresponding encoding and decoding operations, this process ultimately yields the output result for the current pooling kernel. Given that the pooling units can be processed in a pipelined fashion, a theoretical acceleration effect of *d*^2^ can be achieved. The architecture of the pooling acceleration unit is illustrated in Figure 5.

### 3.4. Accelerator Architecture

**Figure 6:**
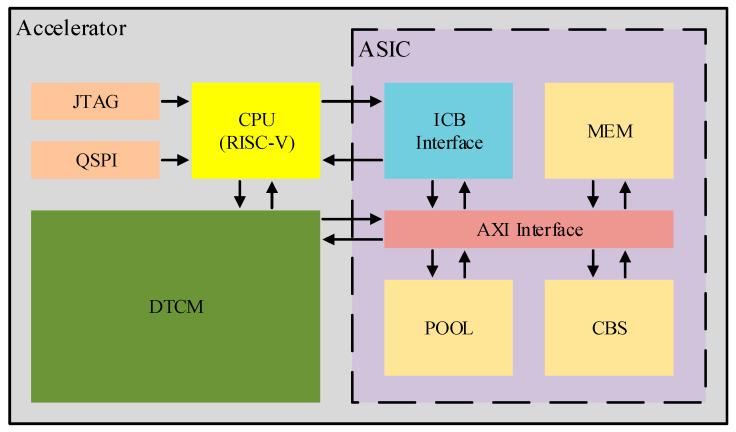
Accelerator architecture. The components are color-coded based on their functional roles: bright yellow for the RISC-V CPU, while dark yellow represents the sub-modules including MEM, POOL, and CBS. The green block denotes the DTCM, orange signifies peripheral interfaces (JTAG and QSPI), and the blue and red blocks indicate the ICB and AXI interfaces, respectively. The purple background identifies the ASIC domain.

The overall architecture of the accelerator is illustrated in Figure 6. The CPU is implemented based on the RISC-V instruction set and features the following functions: (1) fixed-point multiply–accumulate operations, which satisfy the scheduling requirements of the system; (2) a QSPI interface for connecting to the FLASH device that stores parameters such as weights; (3) a JTAG interface for loading control programs to adjust system functionality; (4) the DTCM, which is a CPU-integrated memory module that buffers data generated during the computation process, thereby improving the operational flexibility of the CPU; and (5) an ICB bus, where the CPU interfaces with the ASIC side via an ICB bus to handle the transmission and reception of instructions.

The ASIC side incorporates the following functional modules: (1) ICB bus interface: It interfaces with the ICB bus of the CPU system to facilitate command interaction between heterogeneous systems. (2) CBS: It is a convolution acceleration unit based on a fusion-operator systolic array. (3) Pool: It is a pipeline-based pooling acceleration unit. (4) MEM: It is an independent on-chip RAM module integrated into the ASIC side, which serves as the internal cache of the ASIC.

The primary challenge in heterogeneous computing lies in achieving data synchronization and instruction synchronization across disparate architectures. This architecture addresses this challenge via unified addressing and centralized control mechanisms. Specifically, the ASIC and CPU share a single main memory device (DTCM) with a pre-partitioned address space. During the scheduling phase, the CPU only operates on addresses within the designated data space, thus ensuring consistent data interaction across all architectures. Compared with the traditional data migration scheme, this approach reduces redundant memory accesses and thereby improves overall system performance. Centralized control refers to the strategy where the CPU acts as the master controller to manage the ASIC, which ensures the stable operation of the entire system. Critical feedback data from the ASIC is stored in a dedicated region of the MEM for subsequent retrieval by the CPU, which effectively resolves the cross-clock-domain and instruction conflict issues between heterogeneous architectures.

## 4. Experimental Results and Discussion

### 4.1. Experimental Environment

The accelerator verification platform designed in this paper is implemented on the Digilent Nexys Video development board (Digilent, Inc., Pullman, DC, USA), which is configured with the Xilinx XCA7A35T FPGA chip and operates at a system clock frequency of 100 MHz. The host system is a PC equipped with an Intel Core i5-13600K CPU and an NVIDIA GeForce RTX 3070 GPU, whose main functions include conducting simulation verification of the accelerator and performing training tasks for the network model.

### 4.2. Verification Plan

**Figure 7:**
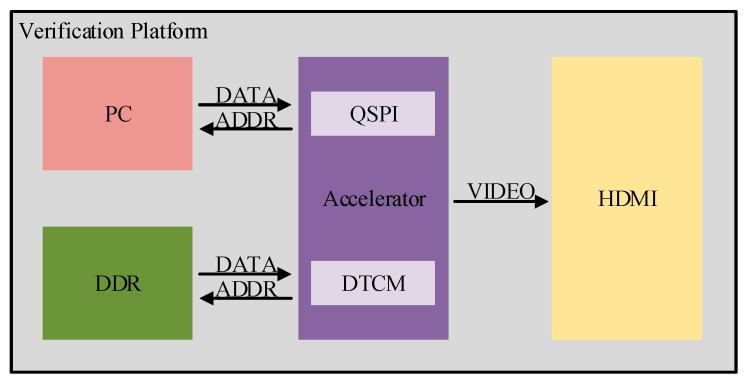
Schematic diagram of the verification platform.

The construction of the accelerator verification platform is illustrated in Figure 7, where the chip designed in this study is marked by the purple box. Since the implementation scheme of this system only supports pre-trained model versions and does not have embedded training capabilities, the DDR simulation method is adopted to emulate external flash devices for inputting parameters such as weights in order to simplify the test platform and reduce testing costs. Meanwhile, an HDMI display device is configured to visually present the output results, thereby enabling an intuitive observation of whether the model outputs are consistent with the expected results.

### 4.3. Performance Evaluation of Operator-Fused Network Models

First, the base model was tested and validated on 889 images from the validation set of BIT-Vehicle, an open-source dataset for vehicle-category classification and detection. The accuracy (P), goodness of fit (R), mean Average Precision at 0.5 (mAP@0.5), and mean Average Precision across IoU thresholds of 0.5 to 0.95 (mAP@0.5–0.95) of the model for each vehicle category were obtained. The validation set consists of 177 SUVs, 545 sedans, 77 microbuses, 38 minivans, 88 trucks, and 39 buses, with the test results presented in Table 2. Subsequently, the operator-fused model was tested on the same validation set of BIT-Vehicle, and the corresponding results are shown in Table 3.

The results indicate that after operator fusion, the model’s accuracy remains nearly unchanged, while its size is reduced to one-third of the original. This not only verifies the effectiveness of the proposed algorithm design but also confirms its adaptability for hardware deployment—an essential requirement in integrated circuit applications.

### 4.4. Performance Evaluation

The resource utilized by the proposed acceleration unit, when deployed on the Nexys Video board platform, is illustrated in Table 4. As can be seen from the data, Block Random Access Memory (BRAM) accounts for the highest utilization rate of 73.84%. By contrast, the flip-flops (FFs), input/output (IO) pins, and global clock buffers (BUFGs) only occupy 2.48%, 3.16%, and 3.13% of the total available resources, respectively. In addition, the utilization rates of look-up tables (LUTs), LUT-based random access memory (LUTRAM), and digital signal processing (DSP) blocks are 8.20%, 6.65%, and 16.62%, which demonstrates a rational allocation of logic computing and dedicated signal processing resources.

To further evaluate the computational performance of the convolutional acceleration unit, a comparative analysis was conducted between its performance and resource consumption and those of other existing FPGA acceleration designs; the comparison results are presented in Table 5.

Even when accounting for parameter discrepancies across implementation schemes, including FPGA device selection, synthesis software configurations, and data type specifications, the proposed design maintains substantial advantages over existing methods. First, it features enhanced functional completeness: integrating convolutional and pooling units addresses the lack of dedicated pooling accelerators in all reference designs, obviating the need to offload pooling operations to external units. Second, CPU collaboration support enables flexible task scheduling by adapting to end-to-end CNN inference requirements. In terms of resource utilization, the design exhibits excellent LUT efficiency with a consumption of only 5.9% of the maximum in the references, boosting applicability to resource-constrained edge FPGA platforms. Moreover, it achieves the highest energy efficiency ratio of 10.46 GOPs/W, significantly outperforming the references. Notably, while maintaining float32 precision for inference accuracy, it delivers a competitive throughput of 20.6 GOPs, realizing a precision–throughput balance suitable for precision-sensitive edge tasks.

However, this design also has certain limitations; its consumption of BRAM is higher than that of the schemes in Reference [41] and Reference [43], which is a design trade-off made for dedicated memory allocation and may restrict the deployment of large-scale models. In addition, the design has limitations in device compatibility, and it requires re-optimization if migrated to high-end FPGA platforms.

## 5. Conclusions

CNN models are evolving toward larger scales and more complex structures. However, this trend poses enormous challenges to the low-power requirements of resource-constrained embedded devices, making it difficult to deploy large-scale CNN models. Existing acceleration solutions either fail to balance computational performance and power consumption or adopt overly complex hardware designs that are incompatible with embedded systems. To address this issue, the CNN accelerator architecture proposed in this paper adopts a heterogeneous computing framework integrating operator fusion and resource optimization strategies, which can effectively alleviate the contradiction between the complexity of CNN models and the resource constraints of embedded devices; maintain high computational efficiency and inference accuracy while ensuring low power consumption; and meet the practical needs of embedded platforms for high-performance and low-power CNN inference.

This paper proposes an FPGA accelerator for the YOLO object detection network. By adopting a heterogeneous computing architecture, the accelerator’s performance is maximized. The final experimental results demonstrate that this solution achieves a computational performance of 20.6 GFLOPs on the Nexys Video board platform, with a power consumption of only 1.96 W, exhibiting improved energy efficiency compared to other FPGA-based accelerator designs. The proposed accelerator architecture achieves approximately 56 frames per second (FPS) during YOLOv5 model inference. Its energy efficiency surpasses both GPU implementations and other large-scale FPGA-based accelerator designs, while its computational efficiency outperforms that of comparable accelerators. This research can be extended to develop specialized computing architectures for computer vision, enabling high-performance and energy-efficient processing of computer vision tasks, thus demonstrating significant potential for practical application.

This advanced technology has broad applicability in numerous edge computing scenarios, all of which have stringent requirements for real-time inference and low power consumption. Specifically, in intelligent security systems, it can support the high-speed processing of surveillance video streams to achieve real-time target detection and behavior analysis, enabling timely responses to potential security threats without relying on cloud computing resources; in the field of autonomous driving, the accelerator allows on-board embedded devices to efficiently run CNN models for environmental perception tasks such as lane detection, pedestrian recognition, and obstacle avoidance, providing reliable computing support for safe driving while meeting the strict power budget of on-board electronic systems; in addition, in wearable devices, its low-power characteristics make it suitable for integration into lightweight hardware such as smart bracelets and smart glasses, realizing functions like real-time health data analysis and gesture recognition without frequent charging, thereby enhancing the user experience.

Nonetheless, the current design still exhibits certain limitations: the throughput remains below that of comparable products, leaving room for further improvement, and the utilization ratio of Block Random Access Memory resources on the FPGA is relatively high. Future work will focus on optimizing hardware resource utilization and expanding support for additional neural network models.

## Figures and Tables

**Table 1:** Network information.

Network	Parameters (M)	Model Sizes (MB)	Release Times
YOLOv3–Tiny	8.7	33.7	2018
YOLOv4–Tiny	6.06	23.1	2020
YOLOv5n	1.9	3.8	2021
YOLOv8n	3.2	6.2	2023

**Table 2:** YOLOv5n model detection results.

Vehicle Category	P/%	R/%	mAP@0.5	mAP@0.5–0.95
SUV	0.929	0.927	0.968	0.918
Sedan	0.983	0.961	0.987	0.931
Microbus	0.93	0.909	0.955	0.911
Minivan	0.943	0.864	0.949	0.866
Truck	0.973	0.989	0.994	0.984
Bus	0.966	1	0.985	0.892

**Table 3:** Model detection performance in this paper.

Vehicle Category	P/%	R/%	mAP@0.5	mAP@0.5–0.95
SUV	0.872	0.913	0.987	0.833
Sedan	0.959	0.954	0.977	0.905
Microbus	0.888	0.753	0.892	0.806
Minivan	0.812	0.68	0.791	0.678
Truck	0.918	0.955	0.964	0.85
Bus	0.913	1	0.968	0.892

**Table 4:** FPGA resource usage.

Resource	Utilization	Available	Utilization/%
LUT	10,975	133,800	8.20
LUTRAM	3072	46,200	6.65
FF	6670	269,200	2.48
BRAM	269.50	365	73.84
DSP	123	740	16.62
IO	9	285	3.16
BUFG	1	32	3.13

**Table 5:** Comparison results of relevant FPGA accelerators.

Parameter		Reference [40]	Reference [41]	Reference [42]	Reference [43]	This Paper
Functional	Convolution Accelerator	√	√	√	√	√
	Pooling Accelerator	×	×	×	×	√
	CPU	×	√	×	×	√
Resource Utilization	LUT	186,251	19,031	32,178	34,995	10,975
	BRAM	1024	25	NA	102	269.5
	DSP	2240	148	125	197	123
FPGA Device		Virtex-VX485t	PYNQ-Z2	Altera 10 GX	ZC702	Artix-7 200T
Operating Frequency (MHZ)		100	100	120	100	100
Data Accuracy		float32	int16	int8	flaot32	float32
Throughput (GOPs)		61.6	23	42.57	5.83	20.6
Energy Efficiency Ratio (GOPs/W)		3.31	5.59	6.5925	2.32	10.46

## Data Availability

The raw data supporting the conclusions of this article will be made available by the authors on request.

## Abbreviations

The following abbreviations are used in this manuscript:

CNN	Convolutional Neural Network
CPU	Central Processing Unit
ASIC	Application-Specific Integrated Circuit
OD	Object Detection
SPPF	Spatial Pyramid Pooling-Fast
BN	Batch Normalization
CONV	Convolution
FPGA	Field-Programmable Gate Array
YOLO	You Only Look Once
IC	Integrated Circuit
SILU	Sigmoid Linear Unit
MaxPool	Max Pooling
CONCAT	Concatenation
CBAM	Convolutional Block Attention Module
CAM	Channel Attention Module
SAM	Spatial Attention Module
DSPs	Digital Signal Processors
MAC	Multiply–Accumulate
PE	Processing Element
BRAM	Block Random Access Memory
FFs	Flip-Flops
IO	Input/Output
BUFGs	Global Clock Buffers
LUTs	Look-Up Tables
LUTRAM	LUT-based random access memory
FPS	Frames Per Second
